# Drug- and/or trauma-induced hyperthermia? Characterization of HSP70 and myoglobin expression

**DOI:** 10.1371/journal.pone.0194442

**Published:** 2018-03-22

**Authors:** Benjamin Ondruschka, Franziska Rosinsky, Heiner Trauer, Eckhardt Schneider, Jan Dreßler, Heike Franke

**Affiliations:** 1 Institute of Legal Medicine, Medical Faculty, University of Leipzig, Leipzig, Germany; 2 Department of Pathology, Group practice Leipzig, Leipzig, Germany; 3 Rudolf Boehm Institute of Pharmacology and Toxicology, Medical Faculty, University of Leipzig, Leipzig, Germany; University of Campinas, BRAZIL

## Abstract

**Introduction:**

Heat shock protein 70 (HSP70) expression could be discussed as an adaption that promotes repair and counteracts cell damage. Myoglobin is released upon muscle damage of several pathways. The purpose of the present study was to determine whether the expression of HSP70 in kidney, heart and brain and of myoglobin in the kidney were associated with the cause of death and the survival times after lethal intoxications with three of the drugs most widely used in our local area (Saxony, Germany) as well as after fatal traumatic brain injury (TBI).

**Methods:**

We retrospectively collected kidney, heart and brain samples of 50 autopsy cases with toxicological proved lethal intoxication (main drugs methamphetamine, morphine, alcohol), 14 TBI cases and 15 fatalities with acute myocardial injury in age- and gender-matched compilations.

**Results:**

Our main findings suggest that HSP70 is associated with hyperthermal and other stress factors of most cell populations. HSP70 expressions in kidney and heart muscle are useful for a differentiation between fatal intoxications and cases without toxicological influence (p < 0.05). There were significant differences in the cerebral expression patterns between methamphetamine- and morphine-associated deaths compared to alcohol fatalities (p < 0.05). An intensive staining of HSP70 in the pericontusional zone and the hippocampus after TBI (especially neuronal and vascular) was shown even after short survival times and may be useful as an additional marker in questions of vitality or wound age. A relevant myoglobin decoration of renal tubules was only shown for methamphetamine abuse in the study presented.

**Conclusion:**

In sum, the immunohistochemical characteristics presented can be supportive for determining final death circumstances and minimal trauma survival times but are not isolated usefully for the detection of drug- or trauma-induced hyperthermia.

## Introduction

There are several mechanisms of death under drug influences, which might be of special forensic interest, if drug concentrations in postmortem blood do not reach lethal doses or questions about potential survival times arises in the evaluation of an individual case. Regarding methamphetamine (METH) abuse there are descriptions of lethal cardiovascular collapse and malignant hyperthermia [1], and reports of METH-related emergency room visits suggest that elevated body temperature is an universal presenting symptom, with lethal overdoses generally associated with extreme hyperthermia [2]. Hyperthermia might therefore be an associated symptom and often a harbinger of death in METH cases [3]. Morphine (MOR) abuse tends to potentially fatal respiratory depression [4] and alcohol (ALC) intoxication to acute right heart failure [5].

Deaths after traumatic brain injuries (TBI) are accompanied by different survival times in accordance with the severity of the impact(s). The primary injuries trigger secondary waves of pathophysiological cascades dependent on the survival times. Since an acute phase response is well-known after TBI in living patients [6], there might be hyperthermal reactions after suffering TBI. In most cases, brain herniation and cerebral dysregulation are the main mechanisms of death after TBI [7], [8].

Hyperthermia is, therefore, an interesting interface of a number of kinds of death. It can promote rhabdomyolysis with release of muscle cell proteins, such as myoglobin, organ failure, increased reactive oxygens and damage cytoskeletal proteins [2].

A variety of chemical and physical cell stress factors (e.g., thermal stress, oxidative stress, ischemic conditions, lack of energy) can induce the expression of heat shock proteins (HSPs) in various cell structures, which were named according to their molecular weight [9]. This family of proteins belong to molecular chaperones, and support cells in their survival attempts in both toxic and lethal conditions [10], [11] and are, therefore, called cytoprotective. In normal conditions, all cells in the body contain such chaperones to manage protein biosynthesis [12] and to fold proteins into their three-dimensional structure. In pathological situations, the HSPs prevent apoptotic cell deaths and induce the repair of damaged proteins [13], [14]. One of the most widely studied HSPs is the ATP-dependent member HSP70 [11, 13], [15], which was the reason for choosing it for the scientific evaluation presented.

Myoglobin is a cytoplasmic, single chain polypeptide found in skeletal and cardiac muscle and functions as an oxygen transporting pigment. Upon muscle damage on several pathways (e.g., rhabdomyolysis), the protein escapes into the environment and is transported until the proximal tubules of the kidneys, leading to so-called crush syndrome [16]. Therefore, the release of myoglobin indicates acute muscle damage with subsequent kidney injury and may be a helpful tool to characterize such potential fatal pathomechanisms.

This report aimed to answer the question, whether there are differences in the detection of HSP70 and myoglobin after potential thermal and/or oxidative stress situations on cells after drug abuse or head injury.

To the best of our knowledge, no combined immunohistochemical studies are available for both proteins in the context of different intoxication patterns and fatal TBI. The purpose of the present study was, therefore, to determine whether the expression of HSP70 in kidney, heart and brain and of myoglobin in kidney were associated with the cause of death and the survival times after lethal intoxications with three of the drugs most widely used in our region as well as after lethal TBI.

## Materials and methods

### Sample collection

This study was approved by the local ethic commission of the Medical Faculty of the Leipzig University (AZ: 117-12-23012012 and 328–08).

Stored samples from the kidney (n = 50), the papillary muscle of the left ventricle of the myocardium (n = 50) and two brain regions, the prefrontal cortex (PFC, n = 50) and the hippocampus (n = 31) were collected retrospectively from 50 autopsy cases with toxicological proven lethal intoxication (n = 16 of METH; n = 16 of MOR; n = 18 of ALC) after fixation in neutral buffered 4% formalin. The period of fixation varied between a few days and four years.

Tissue samples from the kidney and the heart were examined, because both organs had already been investigated with HSP70 and myoglobin for forensic questions [15,17]. Both brain regions, PFC and hippocampus, were also taken to gather information on the time course of potential generalized secondary changes in the central nervous system, such as hypoxia or ischemia and to compare our results in human post-mortem tissue to that of animal experiments [18–20].

The ages of the intoxicated individuals at the time of their death ranged from 22 to 88 years (average age, 42 years). There were 40 males (80%) and 10 females (20%).

Regarding the non-intoxicated groups (all without known peri-mortem thermal influences), 14 cases with lethal TBI and 15 cases with lethal acute myocardial injury (AMI) and survival times between 15 min and 4 h were chosen. Here, samples from the kidney (n = 29), the papillar muscle of the left ventricle of the myocardium (n = 29), the PFC (n = 15; all of AMI), respectively, the pericontusional zone (PCZ) of frontal impact (n = 14; all of TBI) and the hippocampus (n = 19) were collected in the same way. The ages of the non-intoxicated individuals at the time of their death ranged from 21 to 78 years (average age, 53 years). There were 24 males (82.8%) and 5 females (17.2%). Basic characteristics of all case groups are given in detail in **Tables 1 and 2**. Please see electronic supplementary material (**S1 Table**) for more detailed information of single cases.

**Table 1:** Characteristics of the different intoxication groups.

	Gender m: f	Age in y (median; range)	Concentration (median; range)	Addiction yes/no/n.i.	Co-use	Body temperature	Cause of death	PMI in h (median; range)
Methamphetamine-associated deaths (METH)								
n = 16	15: 1	34 (27–46)	1316 ng/ml (275–17.400)	11 / 0 / 5	Negative (n = 10); THC+ (n = 3); morphine+ (n = 2); trenbolone+ (n = 1)	Raised (n = 3); deep (n = 7); n.i. (n = 6)	Mono intoxication (n = 8); mixed intoxication (n = 2); suffocation (n = 3); bleeding (n = 2); hypothermia (n = 1)	63 (10–142)
Morphine-associated deaths (MOR)								
n = 16	11: 5	34 (22–88)	212 ng/ml (110–1246)	16 / 0 / 0	Negative (n = 6); methadone+ (n = 5); alcohol+ (n = 2); benzodiazepine+ (n = 2); barbiturate+ (n = 1)	Raised (n = 2); deep (n = 1); n.i. (n = 13)	Mono intoxication (n = 5); mixed intoxication (n = 8); suffocation (n = 3)	121 (57–159)
Alcohol-associated deaths (ALC)								
n = 18	14: 4	50 (29–79)	0.393% (0.304–0.711)	10 / 3 / 5	Negative (n = 16); morphine+ (n = 1); diphenhydramine (n = 1)	Deep (n = 4); n.i. (n = 14)	Mono intoxication (n = 16); mixed intoxication (n = 1); hypothermia (n = 1)	82 (32–144)

A ‘raised’ body core temperature is stated whenever the postmortem examination showed levels > 37.0°C, and a ‘deep’ temperature whenever the levels were < 37.0°C during measurement. For detailed information please see supplementary materials.

m, male; f, female; y, years; n.i., no information; PMI, postmortem interval; h, hours; +, positive toxicological analysis; THC, tetrahydrocannabinol.

**Table 2:** Characteristics of the non-intoxicated groups.

	Gender m: f	Age in y (median; range)	Survival time (median; range)	Toxicological results	Cause of death	PMI in h (median; range)
Traumatic brain injury (TBI)						
n = 14	14: 0	43 (21–74)	2h 15min (15min–8h)	Negative (n = 10); methamphetamine+ (n = 1); alcohol+ (n = 1); no measurement (n = 2)	TBI (n = 10); polytrauma (n = 4)	58 (10–142)
Acute myocardial injury (AMI)						
n = 15	10: 5	64 (49–86)	‘none’ (n = 7); for rest: 1h 50min (50min–3h)	Negative (n = 11); alcohol+ (n = 1); no measurement (n = 3)	Myocardial infarction (n = 8); coronary insufficiency (n = 3); congestive heart failure (n = 4)	97 (32–144)

Basic characteristics of fatal ‘traumatic brain injury’ and lethal ‘acute myocardial injury’ cases. If ‘none’ is stated as the survival time, the persons collapsed suddenly and died without resuscitation attempts. For detailed information please see supplementary materials.

m, male; f, female; y, years; n.i., no information; PMI, postmortem interval; h, hours; min; minutes; +, positive toxicological analysis.

The post-mortem interval (PMI), i.e. the time interval between the estimated time of death and tissue sampling during autopsy, of all cases ranged from 23 h to 154 h (average PMI, 85 h) with continuously adequate cooling (approximately 4°C).

### Histological and immunohistochemical methods

After different durations of fixation, the tissue samples were embedded in paraffin. Serial sections with a thickness of 6 μm were produced, since this has been shown to offer the best staining results. The sections were deparaffinized in xylene-substitute Neo-Clear for immunohistochemistry (Neo-Clear for immunofluorescence; Merck KGaA, Darmstadt, Germany) and in ALC and were then rehydrated. The tissue sections were firstly stained using hematoxylin and eosin to evaluate the morphology and possible traumatic or inflammatory changes.

Immunohistochemical staining was performed using the standard labelled Streptavidin-Biotin method on AutoStainer plus (DakoCytomation, Glostrup, Denmark).

The antibodies required pretreatment by microwave heating for 20 min, in citrate buffer, pH 6.0 (DakoCytomation). The sections were immersed for 10 min in peroxidase blocking solution (DakoCytomation) to block the endogenous peroxidase.

The primary antibodies used were polyclonal rabbit anti-HSP70 (1:100; Zytomed Systems, Berlin, Germany) and polyclonal rabbit anti-myoglobin (1:50; Zytomed Systems). A biotinylated anti-mouse was used (1:100; DakoCytomation) as a secondary antibody. Diaminobenzidine in horseradish peroxide/phosphate-buffered saline served as a chromogen. Counterstaining was performed with hematoxylin. In addition, careful controls were performed by omitting the primary or secondary antibodies to exclude staining artefacts and to check for non-specific immunoreactions. Tonsillar tissue was used as a positive control for HSP70 and skeletal muscle for myoglobin during the staining procedure.

### Histomorphological evaluation and analysis

Evaluation was conducted microscopically using the Carl Zeiss Axiolab optical microscope (magnification, 50x–400x).

Regarding HSP70, in all cases, the total numbers of tubular cells in the renal cortex, the glomerular podocytes and the Bowman’s capsules were counted in 20 high-power fields (400x magnification) and the positive percentage of these cells were subsequently calculated (Doberentz et al. [11] and Preuß et al. [21] defined these renal cell types to be areas of HSP70 expression). Regarding the brain sections, the total number of neurons, oligodendrocytes, astrocytes and vessel walls in which HSP70 expression was detected in the cortex and the CA1 region of the hippocampus were respectively counted in 10 high-power field, as described previously [22]. Cell classification was performed according to established cytomorphological criteria. We only counted cells which could be allocated accurately to one cell type to ensure the highest possible reliability.

Cardiac muscle fibers and vessels were calculated as proposed previously [11].

A HSP70 percentage > 60% for any of the structures investigated was finally declared as ‘severe’ expression/staining, knowing the typical disadvantages of forensic autopsy material and former data interpretation [11], [21].

Myoglobin staining was used only on kidney samples. The graduation system for the myoglobin staining was presented by Ishigami et al. [17] and gets its classification depending on the extent of reddish-stained tubular structures.

### Double immunofluorescence and confocal microscopy

The treatment procedure for immunofluorescence labeling was performed as described previously [23].

Briefly, the primary antibodies used were polyclonal rabbit anti-HSP70 (Zytomed Systems), mouse anti- microtubule associated protein-2 (MAP2, 1:200; Merck Millipore, Billerica, USA) and mouse anti-glial fibrillary acidic protein (GFAP, 1:1000; Sigma-Aldrich, St. Louis, USA). The simultaneous visualization of different primary antisera was performed with a mixture of secondary antibodies specific to the appropriate species IgG (rabbit, mouse): Carbocyanine (Cy)2- (1:400) and Cy5- (1:100) conjugated IgGs (all Jackson ImmunoResearch, West Grove, USA). Control experiments were performed without primary antibodies.

The multiple immunofluorescence was investigated by a confocal laser scanning microscope (LSM 510 Meta, Zeiss; Oberkochen, Germany) using excitation wavelengths of 488 nm (argon, yellow-green Cy2-immunofluorescence), 543 nm (helium/neon1, red fluorescence) and 633 nm (helium/neon2, blue Cy5-immunofluorescence).

Cy3-labeled antibodies were not used in this study, because of the unspecific auto-fluorescence of lipofuscin with an excitation wavelength of 543 nm (helium/neon1, red fluorescence). Therefore, this wavelength was used as a control for the presence of unspecific lipofuscin autofluorescence in the cells of interest. All red immunofluorescence labeling of different structures shown in the figures prepared resulted from color coding of the Cy5 immunofluorescence to allow a better differentiation between the labeled cells.

### Statistical analysis

Statistical analysis was performed with Microsoft Excel (version 2010; Bellevue, Washington) and the open-source software R (version 3.2.5, 2016), using Spearman’s correlation to calculate the statistical relationship between different structures investigated as well as Fisher’s exact test to examine links between the classification and causes of death. Comparisons between the five groups investigated were made using the non-parametric Kruskal-Wallis test. Post-hoc analyses were performed by means of Dunn test’s, with adjustment by a False discovery rate.

All p values were set as statistically significant at a level of 0.05.

## Results

### Descriptive analyses

The data presented showed an imbalance in the intoxication groups concerning the gender (male cadavers in 94% of METH cases, 69% of MOR cases and 78% of ALC cases) and there were roughly young average ages of the deceased (METH 34 ± 6 years, MOR 34 ± 18 years, ALC 50 ± 13 years). This was the reason for looking for gender- and age-matched non-intoxicated individuals in comparable group sizes. Most cases, especially for MOR, showed a client-typical co-use of other drugs, especially methadone, whereas all lethal ALC cases were mono intoxications without one single exception.

Most of the intoxicated individuals were known for their substance addiction (percentage: METH 69%, MOR 100%, ALC 56%) according to the police records. A ‘first-time’ consumption was not recorded in any of the drug cases.

The survival times of TBI ranged between 15 min and 8 h (median 135 min) and represent ‘acute’ (n = 7) and ‘subacute’ deaths (n = 7) after TBI according to [8] and [22].

Hyperthermia with raised body core temperatures above 37.0°C were diagnosed in three METH cases during external gross examination. Surprisingly, there were even two more cases from the MOR group showing high body temperatures post-mortem. No hyperthermal phases could be detected for any of the other cases.

The PMIs between the case groups are nearly comparable to each other and reflect the width of real circumstances in a German Institute of Legal Medicine.

From a subjective point of view, there was no correlation between the staining quality and the PMI. In addition, there were no relevant correlations between the PMI and the HSP70 expression or the myoglobin expression.

### HSP70 staining

#### Kidney

**Fig 1:**
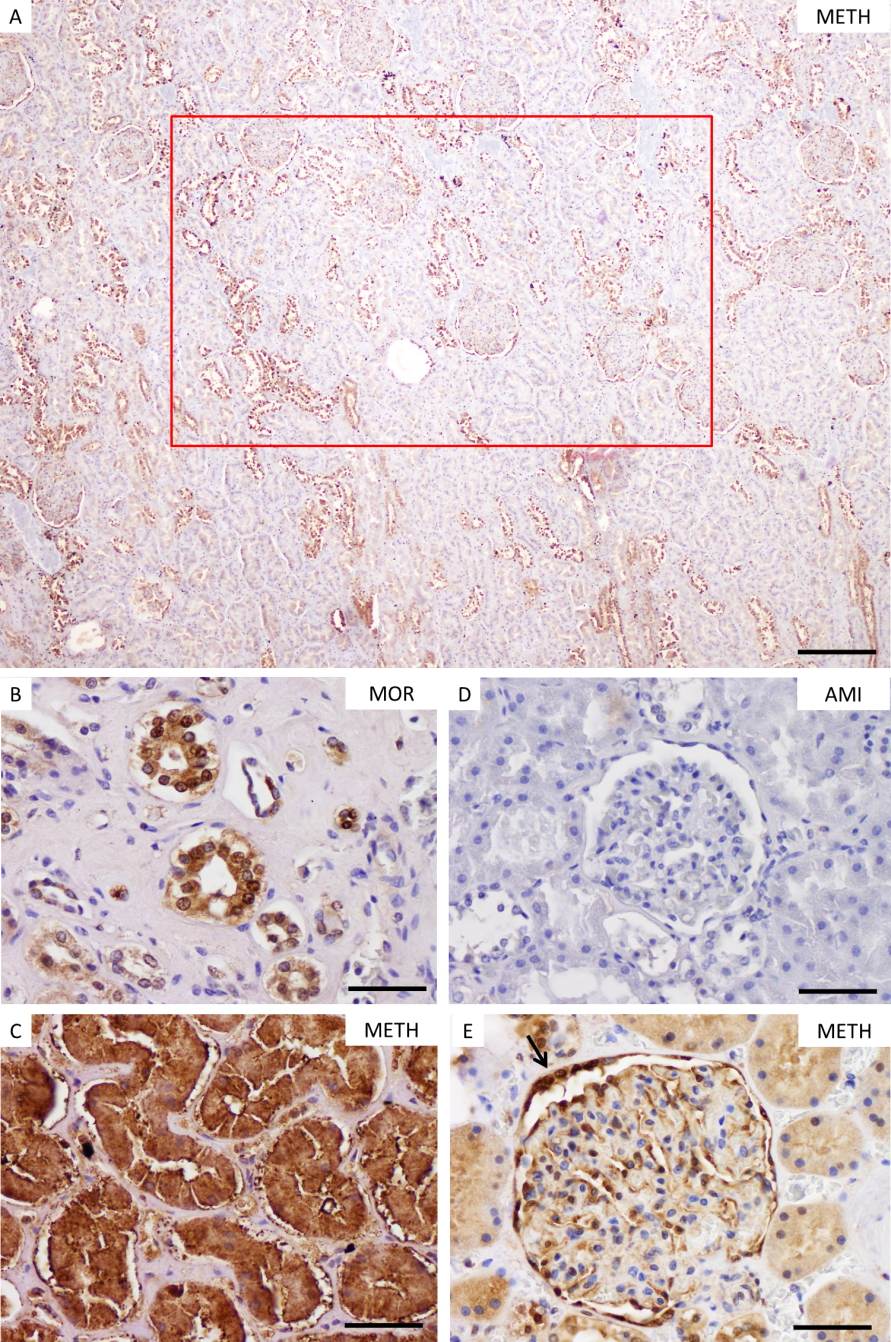
HSP70 staining in kidney samples. Discontinuous HSP70 expression in kidney samples in lower magnification (A, scale bar: 100 μm). The red rectangle represents the counted area per slide with 20 high-power fields. Staining results of the cortex region with partly (B) and all tubular cells (C) stained with HSP70 in intoxication cases. Staining results of glomerular podocytes with immunonegative (D) and -positive staining (E). Please note the immunopositive staining of the Bowman’s capsule in E (thin black arrow). METH, methamphetamine-associated death; MOR, morphine-associated death; AMI, acute myocardial injury. (B-E), scale bar: 25 μm.

The staining behavior of HSP70 generally appeared to be discontinuous in low magnification for kidney sections (see **Fig 1A**). Over 60% of TBI cases showed no HSP70 expression for the tubules. There seemed to be higher percentages of HSP70 positive staining in intoxication groups when compared to non-intoxicated groups (see **Fig 1B–1E**). The results for the renal structures showed statistical differences between intoxicated and non-intoxicated individuals (see detailed description in **Table 3**). Whereas METH, MOR and ALC presented > 50% positively stained structures for glomerular podocytes and Bowman’s capsules (see **Fig 1E**), the non-intoxicated groups showed only negligible percentages in most of the cases. There was a statistically significant correlation between the HSP70 expression in glomerular podocytes and Bowman’s capsules (r = 0.867; p < 0.01). Interestingly, there was even one TBI case with 72% positive stained Bowman’s capsules (*case TBI13*, *see* **S1d Table**). A HSP70 immunopositivity > 60% of all renal structures investigated was detectable in a statistically significant manner for fatal intoxications (p < 0.01), whereas a METH intoxication could not be concluded with certainty (p > 0.05).

**Table 3:** Detailed statistical analysis for HSP70 immunostaining of kidney slides.

	METH, median (IQR)	MOR, median (IQR)	ALC, median (IQR)	TBI, median (IQR)	AMI, median (IQR)	Kruskal-Wallis test	Dunn post-hoc with FDR
**Kidney (n = 79)**							
Tubular cells	36.8 (14.2)	28.5 (16.5)	30.0 (25.5)	2.2 (18.0)	22.5 (18.0)	p = 0.0075	(METH vs. TBI) * (ALC vs. TBI) *
Glomerular podocytes	50.2 (45.9)	66.8 (46.8)	52.5 (55.4)	4.5 (37.5)	27 (42.0)	p = 0.0003	(METH vs. TBI / AMI) * (MOR vs. TBI / AMI) * (ALC vs. TBI / AMI) *
Bowmann’s capsules	72.5 (48.7)	80 (81.2)	57.5 (77.5)	2.5 (22.5)	5 (20.0)	p = 0.0002	(METH vs. TBI / AMI) * (MOR vs. TBI / AMI) * (ALC vs. TBI / AMI) *

Only significant comparisons after a final calculation of false discovery rate (FDR) with adjusted p < 0.05 (*) are presented in the last column.

METH, methamphetamine-associated death; MOR, morphine-associated death; ALC, alcohol-associated death; TBI, traumatic brain injury; AMI, acute myocardial injury; IQR, interquartile range.

#### Heart

**Fig 2:**
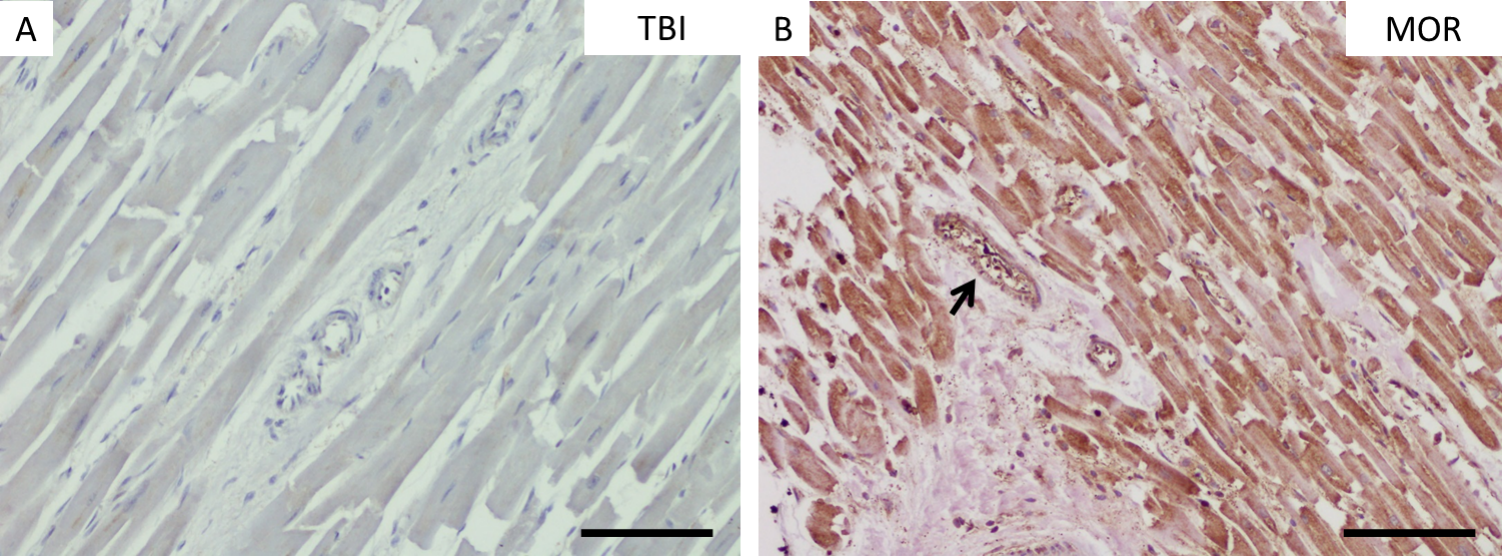
HSP70 staining in myocardial samples. Staining results of HSP70 immunohistochemistry in myocardial muscle samples with immunonegative (A) and -positive staining of the myocytes (B). Please note the immunopositive staining of the intramural vessel walls in B (thin black arrow). MOR, morphine-associated death; TBI, traumatic brain injury. (A-B), scale bar: 50 μm.

The staining behavior of HSP70 was generally nearly continuous in low magnification slices of the hearts (see **Fig 2**). The investigation of HSP70 immunostaining patterns within the heart showed negative results in total for the myocytes in approximately 80% and for the vessels in more than 90% of both groups, TBI and AMI. No single case could be declared as severely stained for the heart samples without proven toxicological influence. Staining intensities for HSP70 did not show any visible differences concerning the drug used.

#### Brain

**Fig 3:**
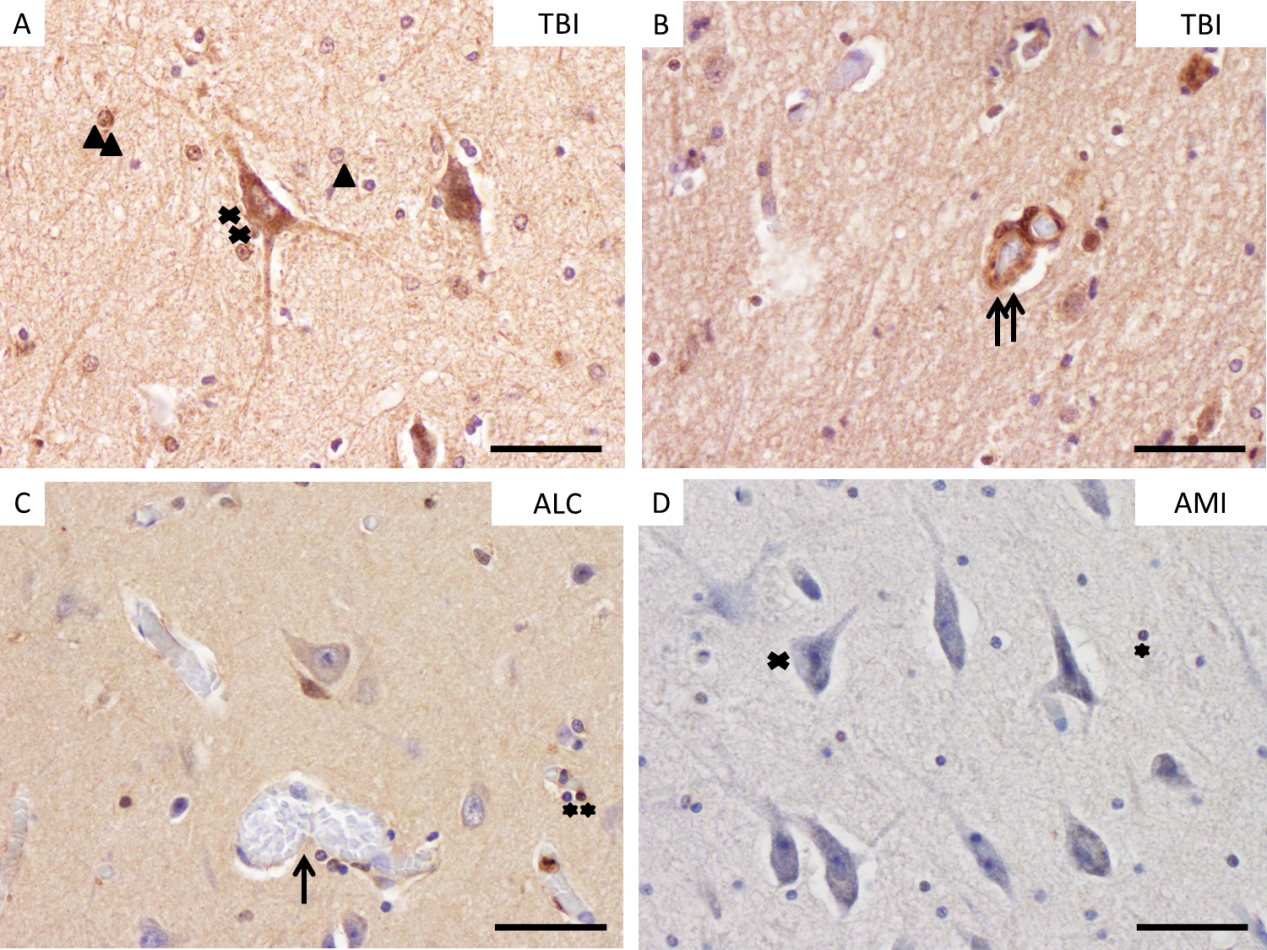
HSP70 staining in brain cortex samples. Staining results of HSP70-immunohistochemistry in brain tissue samples. A: Immunopositive neurons (double cross) and immunopositive and -negative astrocytes (double and single arrowhead) in a pericontusional zone (PCZ) after TBI with a survival time of 40 min. B: Immunopositive vessel walls (double arrow) in a PCZ after TBI. C: Unstained vessel wall (single arrow) and immunopositive oligodendrocytes (double asterisk) in the prefrontal cortex (PFC) of uninjured brain cortex (ALC). D: Immunonegative neuron (single cross) and oligodendrocyte (single asterisk) in the PFC of an AMI fatality. ALC, alcohol-associated death; TBI, traumatic brain injury; AMI, acute myocardial injury. (A-D), scale bar: 25 μm. Please note that every cell type is highlighted only once in this figure with single signs for negative results and double signs for positive staining.

The cell populations within the brain cortex samples were mostly stained in median positive percentages between 20 and 30%. There were only very few cases with > 60% positively stained brain cells out of the METH, MOR and TBI groups (highest single counts: 68.7% of neurons in one MOR case; 75.3% of glial cells in one METH case; 83.9% of vessel walls in one METH case). Please see **Fig 3**with exemplarily pictures of stained cell populations. Statistical differences between the positive cell counts were computed for glial cells and cerebral vessel walls between TBI and AMI cases (see **Table 4A** for tabulated data).

**Table 4:** Detailed statistical analysis for HSP70-immunostaining of brain tissue slides.

	METH, median (IQR)	MOR, median (IQR)	ALC, median (IQR)	TBI, median (IQR)	AMI, median (IQR)	Kruskal-Wallis test	Dunn post-hoc with FDR
**A)**							
**Brain—prefrontal cortex (n = 79)**							
astrocytes	23.4 (31.1)	25.2 (25.4)	23.8 (13.9)	35.1 (17.6)	17.5 (19.5)	p = 0.0075	(TBI vs. AMI) *
oligodendrocytes	25.0 (25.3)	20.8 (17.3)	20.1 (9.6)	24.2 (11.4)	12.3 (13.4)	p = 0.0155	(TBI vs. AMI) *
neurons	35.1 (36.1)	41.6 (28.6)	24.2 (13.3)	45.8 (17.9)	29.7 (38.5)	p = 0.0427	none
vessel walls	26.9 (17.7)	30.6 (31.0)	17.1 (4.6)	35.5 (30.8)	20.0 (26.0)	p = 0.0063	(TBI vs. ALC / AMI) *
**B)**							
**Brain–hippocampal CA1 region (n = 50)**							
astrocytes	18.5 (27.9)	24.1 (41.8)	9.2 (14.8)	27.9 (24.2)	13.7 (10.4)	p = 0.0147	(MOR vs. ALC) * (TBI vs. ALC) *
oligodendrocytes	30.7 (32.0)	21.9 (42.3)	5.5 (9.6)	27.5 (22.0)	18.8 (5.9)	p = 0.0049	(METH vs. ALC) * (MOR vs. ALC) * (TBI vs. ALC) *
neurons	18.7 (35.9)	21.8 (35.1)	2.6 (4.3)	43.9 (39.4)	22.1 (18.8)	p = 0.0032	(TBI vs. ALC) *
vessel walls	52.0 (47.7)	19.4 (20.2)	7.4 (17.4)	40.5 (23.5)	19.0 (33.3)	p = 0.0015	(METH vs. ALC) * (TBI vs. ALC) *

Only significant comparisons after a final calculation of false discovery rate (FDR) with adjusted p < 0.05 (*) are presented in the last column.

METH, methamphetamine-associated death; MOR, morphine-associated death; ALC, alcohol-associated death; TBI, traumatic brain injury; AMI, acute myocardial injury; IQR, interquartile range.

The results concerning the positive percentages of cell types within the CA1 region of the hippocampus formation are shown in **Table 4B**. The highest positive HSP70 levels were present in METH and TBI cases, whereas the lowest median levels were counted for ALC intoxicants. The number of positive numbers is higher than in glial cells in both brain regions after TBI.

**Fig 4:**
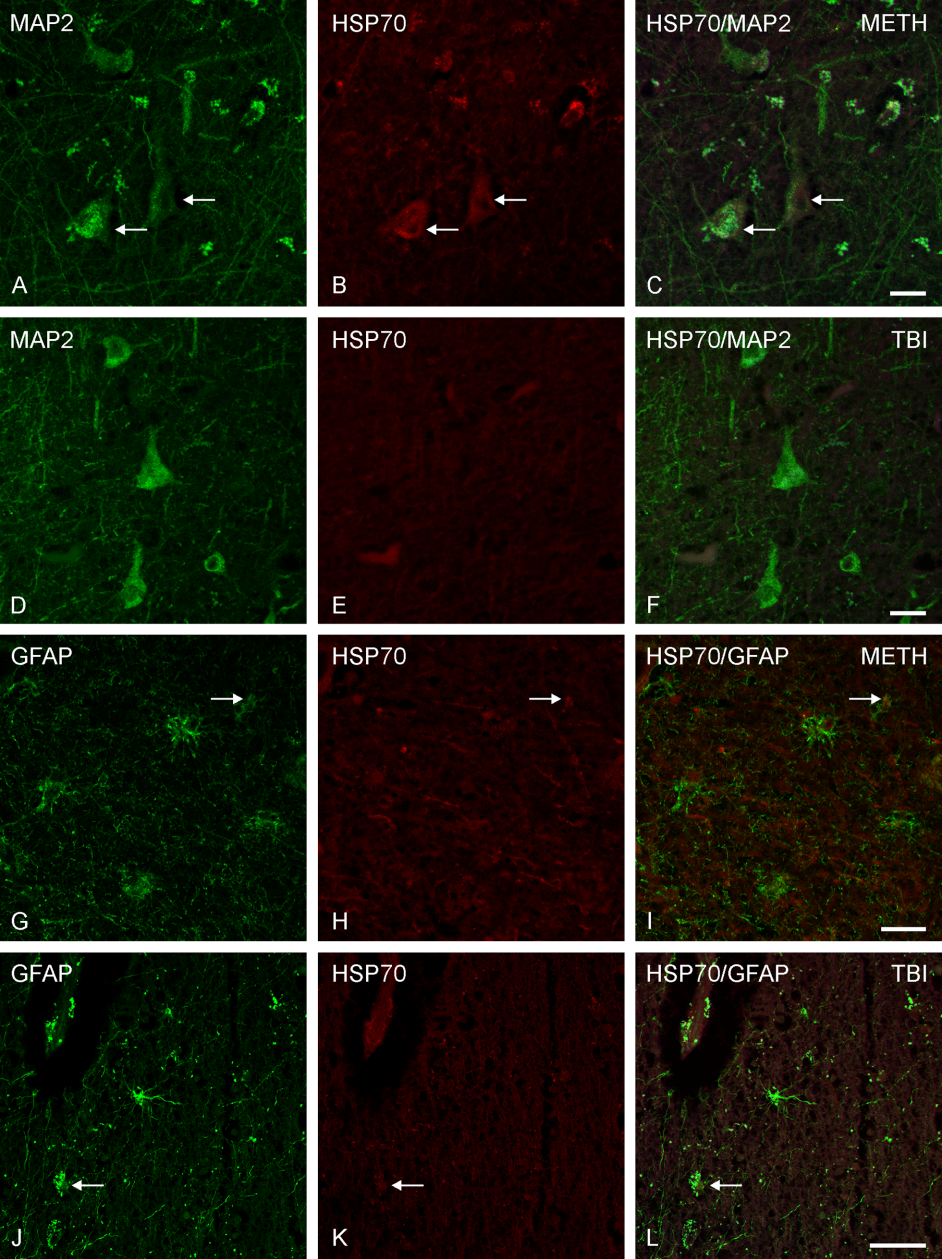
Double immunofluorescence in brain cortex samples. Representative HSP70-positive stained neurons and astrocytes in brain cortex samples using immunofluorescence methods with confocal microscopy. (A-C) Two HSP70 and MAP2 positive multipolar neurons (arrows) with double labeling; (G-I) One astrocytic double labeling of HSP70 and GFAP (arrow); both rows: Methamphetamine (METH)-associated death, scale bar: 20 μm. (D-F) No neuronal co-expression of MAP2 and HSP70. The cause of death was traumatic brain injury (TBI), scale bar: 20 μm. (J-L) Weak double labeled astrocyte by GFAP and HSP70 (arrow). TBI case, scale bar: 50 μm.

In order to confirm the results of the brain samples and to rule out staining artefacts, double immunofluorescence labeling was performed, where we approved positively labeled cells to be of neuronal origin by MAP2 immunostaining and to be of glial origin by GFAP immunostaining (see **Fig 4**). Furthermore, there seemed to be an influence on HSP70 staining associated to apoptotic or aging processes of cells within the brain tissue (Lipofuscin-positive).

**Fig 5:**
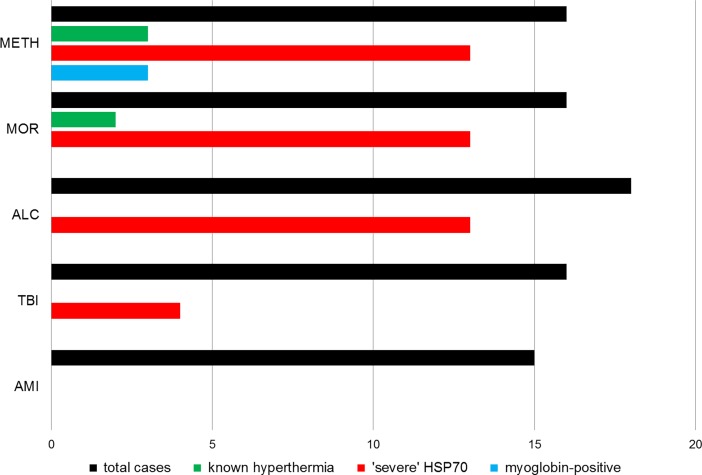
Summarized case data. Comparison of total case numbers per group (black) with percentage of known hyperthermal phases (green) and overall ‘severe’ HSP70 staining in any of the organs investigated (positive percentages > 60%; red) or myoglobin positive staining of the kidney (blue). METH, methamphetamine-associated death; MOR, morphine-associated death; ALC, alcohol-associated death; TBI, traumatic brain injury; AMI, acute myocardial injury.

In sum, 81% of all METH cases, 81% of all MOR cases, 72% of all ALC cases and 29% of all TBI cases showed a ‘severe’ HSP70 expression/staining (defined by [11] and [21] as HSP70 positive percentage > 60%) in at least one organ structure investigated, whereas AMI cases did not show any relevant HSP70 staining reaction at all (see **Fig 5**). Therefore, AMI and comparable sudden natural death cases with cardiovascular collapse could be declared a suitable control when using HSP70 immunohistochemistry.

### Myoglobin staining

**Fig 6:**
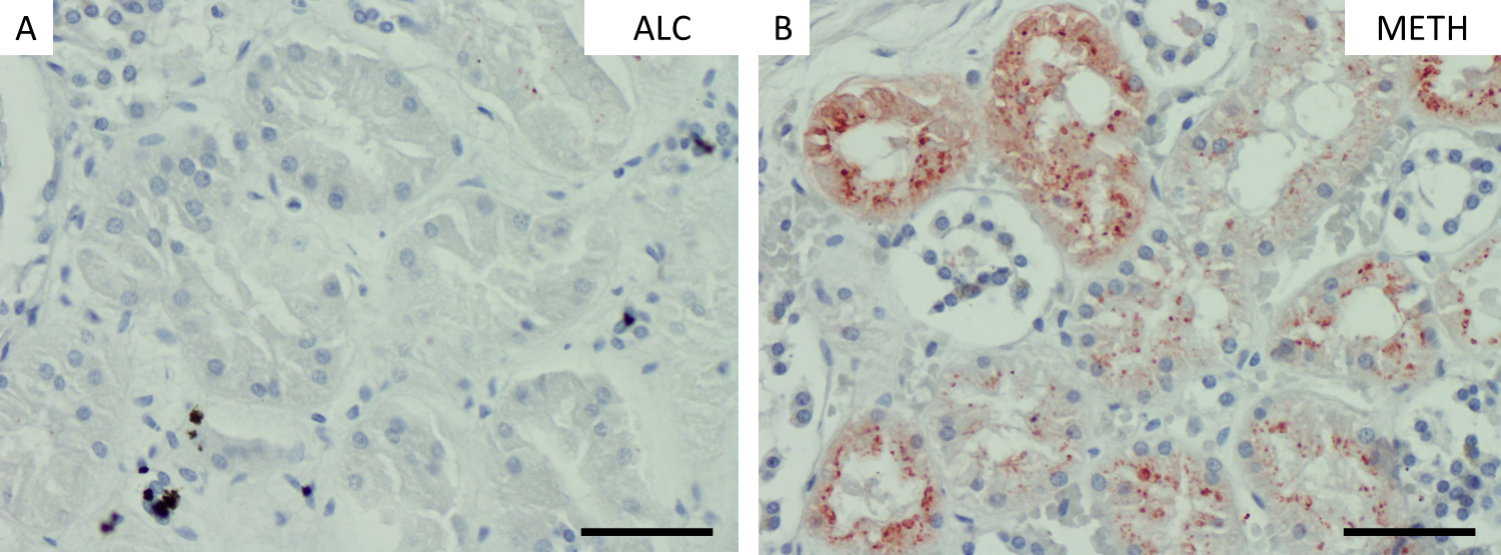
Myoglobin staining in kidney samples. Staining results of myoglobin immunohistochemistry in kidney samples with immunonegative (A) and -positive tubular staining (B). METH, methamphetamine-associated death; ALC, alcohol-associated death. (A-B), scale bar: 25 μm.

Only METH-cases showed strong positively results using myoglobin immunohistochemistry when compared to the other groups collected (p < 0.01) with such myoglobin positive staining in one fifth of all METH cases (see **Fig 5**). This result tended to be a sign of rhabdomyolysis. The nonintoxicated groups showed negative staining results (see **Fig 6**). There were no glomerular myoglobin deposits.

### Expression according to drug blood concentrations

It is of interest, that all three myoglobin-positive METH cases present positive percentages > 60% for some of the kidney structures investigated by HSP70 immunohistochemistry. We were not able to show any correlation between the blood concentration of METH (maximum r = 0.331, p = 0.20) or MOR (maximum r = 0.146, p = 0.59) and the staining patterns of HSP70 in the kidney.

Both ALC cases with the highest blood ALC content measured showed positive percentages > 60% for renal structures and a severe staining pattern in the heart. Correlation calculations for the concentrations measured were waived, since all the other blood ALC contents were nearly comparable > 0.3%.

In sum, there were significant differences in the cerebral expression patterns between METH- and MOR-associated deaths compared to ALC fatalities (p < 0.05).

### Expression according to thermal effects

All three METH cases with proved hyperthermia were HSP70-positive in the heart, showed positive percentages > 60% in the kidney in two cases and were even myoglobin-positive in two cases. Both MOR cases with raised temperature profiles showed a comparable result for HSP70-staining but not for the myoglobin reaction. The explanation for the hyperthermal reaction in the case of MOR6 seemed to be associated with a so-called junky pneumopathia with a fever reaction of the body due to chronic inflammatory processes.

### Expression according to trauma survival times

The PCZ of the injured brain samples showed positive staining percentages above the threshold of 60% of neurons and vessels after survival times of 15 min, 30 min and 4 h *(cases TBI1*, *TBI3*, *TBI9* respectively; *see* **S1d Table**), but never for glial cells (highest count for astrocytes: 51.4% positive; highest count for oligodendrocytes: 34.7% positive). Interestingly, the positive percentage of neurons in the hippocampus exceeded 60% in one further trauma case after 2 h 30 min survival *(case TBI8; see* **S1d Table**). Additionally, the maximum counts mentioned were not reached for glial cell groups in the CA1 region of the hippocampus formation as well, but median levels differed significantly between TBI and ALC cases for both astrocytes and oligodendrocytes in the hippocampus.

## Discussion

The aim of this study was to establish an additional tool for daily casework in forensic medicine in the form of an immunohistochemical investigation of lethal intoxications and fatal TBI to determine the manner and circumstances of death in such cases using the proteins HSP70 and myoglobin. We wanted to answer the question whether there are any drug-dependent and trauma-induced mechanisms leading to fatal hyperthermal body reactions.

Our main findings show that HSP70 expressions in kidney and heart muscle are useful for a differentiation between fatal intoxications and cases without toxicological influence. An intensive staining of HSP70 in the PCZ and the hippocampus after TBI was shown even after short survival times and may be useful as an additional marker in questions of vitality or wound age. A relevant renal myoglobin decoration was only shown for METH abuse.

Therefore these results suggest that HSP70 is associated with hyperthermal and other stress factors of most cell populations [9] or even to non-stressful conditions [24]. Strong HSP70 expression could be viewed as an adaption that promotes repair and counteracts cell damage, as was stated previously [25].

### Drug-induced expression of HSP70 and myoglobin

Deaths associated with drug abuse can pose a forensic challenge at a crime scene investigation when external signs of consumption, for example fresh intravenous punctures, injecting equipment or crystalline-like substances are missing [26]. Although a history of drug abuse by the deceased is known from both anamnestic data or police records in most cases, an acute influence of illicit drugs can only be proofed by forensic autopsy and subsequent toxicological analyses. The use of METH is becoming increasingly popular in our local region in East Germany [27] and seems to be pushing all other illicit drugs out of the ‘market’ locally.

Knowing the fact that one of the main problems in METH intoxications is an uncontrolled heating of the body (central hyperthermia), the question arose whether this phenomenon existed in every relevant METH influence, such as Matsumoto et al. [2] hypothesized, or whether this is only one of several potential life-threatening mechanisms of METH pathology.

Our results indicate, firstly, that not every METH intoxication is associated with elevated body temperature and, secondly, especially high METH concentrations should be fatal, causing severe sympathomimetic effects, such as tachycardia and raised blood pressure rather than using the hyperthermal pathway. Ishigami et al. [17] stated a relevant association between METH consumption and immunopositive myoglobin staining of kidney slices in their report, with 23% strongly positive cases, which is a quite good comparison with the results presented here (19%). As our results indicate, Ishigami et al. [17] did not report any glomerular staining using myoglobin, which is explained best by the penetrability of the glomerular podocytes for the protein. Thus, the myoglobin staining results might underpin the hypothesis, causing relevant muscle damage due to shivering caused by raised body temperature, and seemed to be associated with prolonged agony phases of death, which is explainable by regular and repeated consumption rather than heavy single doses of METH [28]. Of course, muscle protein staining in kidneys of drug abusers is not associated with METH alone, as there are post-mortem and clinical case reports on MOR and ‘ecstasy’ intoxications as well [29–31]. Nonetheless, METH abuse is becoming an increasingly common cause of rhabdomyolsis in clinical settings [32].

While Ishigami et al. [17] reported only 5 out of 22 cases with HSP70-positive kidney staining and, therefore, fewer than presented here, the main differences between the investigations could be the usage of other antibodies, different counting systems (they used semi-quantitative counting only) and especially huge discrepancies in the mean METH blood concentration (9,900 ng/ml vs. 2,900 ng/ml). However, the rate of paired HSP70- *and* myoglobin-positive kidney staining for a single case in the METH group was strongly comparable to each other in both reports. This result may be interpreted as being an oxidative cell stress caused by the myoglobin accumulation in the kidney tissue, the more prominent factor tending to a HSP70 expression rather than the hyperthermia itself [17]. When stated conservatively, at least an influence of several noxious agents, not only a direct hyperthermal effect seemed to be associated to an expression of HSP70 for cell protection.

HSP70 and other examples of the HSP family (especially HSP27) are widely investigated by the working group Doberentz et al. [11], [15], [33], [34]. They presented results about HSP70 expression in cases of fatal hyperthermal effects such as burns and fire accidents [11], with a special focus on the expression pattern in different agony phases [15], sudden infant death syndrome [33] and defined a ‘semi-quantitative graduation’ for this staining first in 2008 while investigating fatal hypothermia [21]. We decided to explicitly go for an evaluation of HSP70 behavior alone, instead of HSP27 as well, since the latter is known as an ATP-independent chaperone [35].

Whereas temperature profiles in cases of burning are much higher than in intoxication or trauma cases, the Doberentz working group referred to HSP70 expression in renal tubular cells independent of the survival time and stated this expression pattern to be at least a clear sign of vitality [11]. According to our results, a severe HSP70 expression in the kidney and/or the heart could be expected whenever a documented heat stress has been proven, no matter whether this effect was due to external or internal influences. In a conference paper aimed to investigate HSP expression patterns in 20 amphetamine-associated fatalities the same working group did not show any HSP70 reaction, which contradicts all other publications cited [36]. Nevertheless, even fatal hypothermia is known to influence the HSP70 expression in human kidneys [21] and cerebral cortex of rats [37], which showed a nearly ubiquitous HSP70 expression due to several stress stimuli on cells rather than hyperthermal effects alone.

The positive staining of HSP70 in myocardial tissue after METH intake was reported by Tomita et al. [38]. We argued to interpret this cardiac HSP expression in a form of a cardioprotective behavior after stress stimuli. However, there seemed to be no general ischemic-specific expression in myocytes after myocardial infarction with respect to our results. Obviously, this may be explained partly by collecting samples of the papillary muscle of the left ventricular wall and not out of the infarction area. Dybdahl et al. showed elevated blood HSP70 concentrations after an AMI [39]. Additionally, raised body temperatures within 48 h after an infarction could be shown [40]. It seemed plausible that such elevated temperatures take place during healing processes, for example remodeling of the heart muscles, but not within the agony phase during fatal cases.

While Kuperman et al. [19] presented qualitatively positive HSP70-expressions in the cortical cortex and hippocampus samples of mice after METH intake, our results from the hippocampal samples of the intoxication groups showed significant median differences for positive HSP70 counts for METH compared with ALC cases (oligodendrocytes, vessel walls).

The preference of HSP70 immunopositive vessels presented in different organs has also been described previously and may indicate that hyperthermal and other stress stimuli occur immediately before death [1], [11], [18]. Another explanation might be that the HSP70 expression protects cells that comprise the vasculature against cell death owing to proinflammatory responses [41].

To the best of our knowledge, little to nothing is known concerning hyperthermal reactions after MOR intake in humans. On the other hand, this phenomenon was described in rats before [42] and as a withdrawal symptom in vivo [43], while there was a marked increase of HSP70 in neurons and cerebral blood vessels in response to MOR in rats [44]. The latter may indicate neuroprotective issues rather than the direct consequence of heat exposure. We could show such results for glial cells in MOR fatalities when compared to ALC cases. Interestingly, HSP70 has been described before for protective effects after ALC intake [45], [46]. This might be one explanation, why fatal cases showed very low HSP70-positive cells in the hippocampus in this study.

### Trauma-induced expression of HSP70

Mild hyperthermal reactions were described as symptoms of a TBI and are known to be a dangerous complication during intensive care within the secondary brain damage after injury, called central fever [47]. This may explain the positive staining in some cases, although the temperature level is regulated by the hypothalamic region, which was not investigated here. According to the results presented here, intensive pericontusional HSP70 staining was shown after mechanical damage in rabbits’ brains [48]. Indeed, intensive HSP70 staining in such regions may indicate a minimal trauma survival of at least 15 min and may, therefore, be used as an additional marker for vitality and early wound age estimations after fatal TBI in addition to established proteins [22]. Our data may support the hypothesis that HSP70 induction is involved in neuroprotection against subsequent ‘secondary’ injury (see [49] for a review). Further research is necessary to show whether longer TBI survival will result in more intensively HSP70 stained brain cells by HSP70. Perhaps there will be a biphasic response of glial cells after the time period investigated, knowing that they are late in their response to cellular stress forms [19]. On the other hand, Goto et al. did show even faster glial expression of the protein in mice after METH induction [18].

The only TBI case with HSP70 positivity > 60% in the kidney has to be declared as special, since the deceased showed a relevant METH blood concentration.

Nevertheless, questions have often arisen whether such fast expressions as presented are plausible on a protein level, but Doberentz et al. [15] demonstrated HSP expression even within seconds and minutes after heat exposure. The survival time had an influence on the HSP expression [15], underlining the validity of the results presented. Furthermore, a fast HSP70 expression in a high percentage of neurons after ischemic cell damage with an expression decrease over time has been described in animal experiments before [50]. Hecker and McGarvey reviewed that the release, transcription and translation of HSPs are among the fastest of intracellular severe stress responses [51]. Additionally, our working group could even show a link between HSPA12B mRNA expression, encoding HSP70 and the influence of hypoxic periods on TBI severity [52] with significant discriminations in expression pattern between TBI and AMI cases. Furthermore, Michael et al. declared HSP70 to be immediately upregulated in the PCZ of TBI patients by gene expression microarray [53]. The next research step could be a biochemical assessment of serum and/or cerebrospinal fluid levels of HSP70 in different causes of death, since it was described as an early predictor of fatal outcome after severe TBI in humans [54]. Furthermore, Western blot analyses might show changes in protein levels of the organs investigated, and this remains to be carried out in the future. Unfortunately, neither biochemical nor Western blot analysis were possible for our cohort samples due to the retrospective study design and local storage regulations.

### Limitations

Concerning both proteins HSP70 and myoglobin, there was a rather wide range of expression even within one case group. This might be due to the fact of the existing heterogeneity of the corpses to be observed as well as the samples’ different PMIs. Next, we were not able to exclude potential influences of co-use of other drugs on the staining behavior and there are wide ranges of the drug concentrations per intoxication group.

Most of the intoxicated individuals were known for their chronic substance intake. The influence of regular use up to addiction towards the expression pattern of HSP70 has been unknown until now and needs to be answered in the future to get a detailed insight into the potential effects of chronic abuse. Two animal experiments were conducted for duration times of HSP70 expression after single exposure to heat stress and the results indicate organ-dependent expression durations within a few hours [55], [56]. If an HSP70 expression could also be associated to a chronic substance abuse also and not solely to the acute intake, immunopositive reactions in organs of interest would tend to indicate at least the addiction behavior of the last few days. Investigations in mice showed a temperature rise of about 2.6°C after a single METH injection after 1.5 h and a return to control values 3 h after injection [19].

Therefore, a further problem might be the time point of external postmortem investigation and therefore the declaration if any hyperthermal reaction was established. It is not entirely clear whether any of the deceased with ‘normal’ (around 37°C) or ‘deep’ (< 37°C) rectal temperatures during measurement ever showed a hyperthermia during agony. Such problems would only be eliminable if there had been consecutive measurements of the body temperature in witnessed death cases, perhaps in intensive care units, but this requires a systematic prospective study setting.

Correlations between HSP70 staining, the duration of storage of the tissues and, therefore, the age of the sample slices could not be proved by us or others [11], [17], [21], [33], which may declare the protein as feasible for retrospective investigations. However, a possible influence on denaturation processes with long storage periods cannot be totally excluded.

In the next step, other proteins of the HSP family could be investigated, such as HSP27, to get further insights into the expression behavior of HSPs, especially to investigate if this prototype of ATP-independent HSP will result in other (faster?) expression after toxic or traumatic stress stimuli.

## Conclusion

Immunohistochemical investigations of HSP70 expression in kidney and heart muscle are useful for a differentiation between fatal intoxications and cases without toxicological influence. The results do not allow an assignment to a specific drug, although only METH is known to be associated with hyperthermal reactions in clinical routine at the present time.

A relevant myoglobin decoration of renal tubules was only shown for METH cases in the study presented.

Elevated HSP70 expression after TBI were shown in the PCZ and the hippocampus even after short survival times and may be useful as an additional marker in questions of vitality or wound age.

In sum, the immunohistochemical characteristics presented can be supportive for determining final death circumstances and minimal survival times but are not useful in isolation for survival times of 15 min, 30 min and 4 h the detection of drug- and/or trauma-induced hyperthermia.

## Supporting information

S1 Table Detailed characteristics of all deceased divided by their causes of death. (DOC)

## Abbreviations

ALC: Alcohol
AMI: Acute myocardial injury
HSP: Heat shock protein
METH: Methamphetamine
MOR: Morphine
PCZ: Peri-contusional zone
PFC: Prefrontal cortex
PMI: Post-mortem interval
TBI: Traumatic brain injury
