# Supplementary material for: Drug- and/or trauma-induced hyperthermia? Characterization of HSP70 and myoglobin expression
Source: PLoS One. 2018 Mar 22;13(3):e0194442. doi: 10.1371/journal.pone.0194442 (PMC5864017; doi:10.1371/journal.pone.0194442)
Supplement: S1 Table — (DOC) [file pone.0194442.s001.doc]

|  | **Gender** | **Age** | **Concentration (in ng/ml)** | **Addiction** | **Co-use** | **Temperature** | **Cause of death** | **PMI (in h)** |
| --- | --- | --- | --- | --- | --- | --- | --- | --- |
| **METH1** | m | 28 | 3.100 | yes, month | negative | 39.5 °C  (2 hpm) | Intoxication | 31 |
| **METH2** | m | 28 | 558 | yes, month | negative | deep | Suffocation | 95 |
| **METH3** | m | 43 | 293 | unknown | negative | deep | Hypothermia | 59 |
| **METH4** | m | 37 | 275 | unknown | negative | n.m. | Suffocation | 57 |
| **METH5** | m | 33 | 1.336 | yes, years | THC+ | n.m. | Intoxication | 68 |
| **METH6** | m | 27 | 836 | yes, years | Trenbolone+ | n.m. | Suffocation | 10 |
| **METH7** | m | 33 | 17.400 | yes, month | negative | n.m. | Intoxication | 23 |
| **METH8** | m | 46 | 2.718 | yes, years | *MOR 197 ng/ml* | n.m. | Mixed intoxication | 118 |
| **METH9** | m | 36 | 1.296 | yes, month | negative | deep | Intoxication | 142 |
| **METH10** | f | 27 | 425 | yes, month | THC+ | deep | Aortal dissection | 98 |
| **METH11** | m | 36 | 545 | unknown | negative | deep | Bleeding | 55 |
| **METH12** | m | 35 | 4670 | yes, years | THC+ | n.m. | Intoxication | 74 |
| **METH13** | m | 32 | 907 | unknown | *MOR 104 ng/ml* | 38.2 °C  (2.5 hpm) | Mixed intoxication | 86 |
| **METH14** | m | 44 | 3957 | unknown | negative | 40.1°C (0.5 hpm) | Intoxication | 41 |
| **METH15** | m | 29 | 4490 | yes, years | negative | deep | Intoxication | 82 |
| **METH16** | m | 42 | 3000 | yes, years | negative | deep | Intoxication | 34 |

**S1a Table.**  Characteristics of the deceased with methamphetamine-associated death.

PMI, postmortem interval; h, hours; METH, methamphetamine; m, male; f, female; +, positive toxicological analysis; THC, tetrahydrocannabinol; MOR, morphine; hpm, hours postmortem; deep; body core temperature <37.0 °C during postmortem examination; n.m., no measurement.

|  | **Gender** | **Age** | **Concentration (in ng/ml)** | **Addiction** | **Co-use** | **Temperature** | **Cause of death** | **PMI (in h)** |
| --- | --- | --- | --- | --- | --- | --- | --- | --- |
| **MOR1** | f | 88 | 133 | yes, years | Barbiturate+ | n.m. | Mixed intoxication | 130 |
| **MOR2** | m | 24 | 111 | yes, years | Methadone+ | deep | Mixed intoxication | 57 |
| **MOR3** | m | 31 | 180 | yes, years | Diazepame+ | n.m. | Suffocation | 121 |
| **MOR4** | m | 35 | 121 | yes, years | *ALC 0.140 %* | n.m. | Mixed intoxication | 90 |
| **MOR5** | m | 26 | 200 | yes, months | Flunitrazepame+ | 38.1 °C (2.0 hpm) | Suffocation | 106 |
| **MOR6** | m | 39 | 670 | yes, years | negative | 40.1 °C (0.5 hpm) | Intoxication | 122 |
| **MOR7** | m | 34 | 295 | yes, years | negative | n.m. | Intoxication | 127 |
| **MOR8** | f | 27 | 634 | yes, years | Methadone+ | n.m. | Mixed intoxication | 94 |
| **MOR9** | f | 37 | 184 | yes, years | Methadone+ | n.m. | Mixed intoxication | 128 |
| **MOR10** | f | 55 | 427 | yes, years | negative | n.m. | Suffocation | 148 |
| **MOR11** | m | 43 | 144 | yes, years | negative | n.m. | Intoxication | 144 |
| **MOR12** | m | 73 | 366 | yes, years | negative | n.m. | Intoxication | 159 |
| **MOR13** | m | 25 | 370 | yes, years | Methadone+ | n.m. | Mixed intoxication | 94 |
| **MOR14** | f | 33 | 225 | yes, years | Methadone+ | n.m. | Mixed intoxication | 98 |
| **MOR15** | m | 36 | 1246 | yes, month | *ALC 0.180 %* | n.m. | Mixed intoxication | 126 |
| **MOR16** | m | 22 | 110 | yes, years | negative | n.m. | Intoxication | 119 |

**S1b Table.**  Characteristics of the deceased with morphine-associated death.

PMI, postmortem interval; h, hours; MOR, morphine; m, male; f, female; +, positive toxicological analysis; ALC, alcohol; hpm, hours postmortem; deep; body core temperature <37.0 °C during postmortem examination; n.m., no measurement.

|  | **Gender** | **Age** | **Concentration (in %)** | **Addiction** | **Co-use** | **Temperature** | **Cause of death** | **PMI (in h)** |
| --- | --- | --- | --- | --- | --- | --- | --- | --- |
| **ALC1** | f | 42 | 0.304 | unknown | negative | n.m. | Intoxication | 32 |
| **ALC2** | m | 51 | 0.419 | unknown | negative | n.m. | Intoxication | 72 |
| **ALC3** | f | 40 | 0.341 | yes, years | negative | n.m. | Intoxication | 144 |
| **ALC4** | m | 50 | 0.397 | yes, years | negative | deep | Hypothermia | 92 |
| **ALC5** | m | 42 | 0.406 | yes, years | negative | n.m. | Intoxication | 76 |
| **ALC6** | m | 67 | 0.336 | yes, years | negative | n.m. | Intoxication | 86 |
| **ALC7** | m | 56 | 0.339 | unknown | negative | n.m. | Intoxication | 86 |
| **ALC8** | m | 35 | 0.495 | yes, years | negative | n.m. | Intoxication | 80 |
| **ALC9** | m | 41 | 0.444 | unknown | negative | deep | Intoxication | 106 |
| **ALC10** | m | 71 | 0.348 | yes, years | negative | n.m. | Intoxication | 36 |
| **ALC11** | m | 38 | 0.446 | unknown | negative | n.m. | Intoxication | 73 |
| **ALC12** | m | 58 | 0.427 | yes, years | negative | n.m. | Intoxication | 59 |
| **ALC13** | m | 72 | 0.389 | yes, years | negative | deep | Intoxication | 122 |
| **ALC14** | m | 29 | 0.365 | yes, years | *MOR 28 ng/ml* | n.m. | Intoxication | 84 |
| **ALC15** | f | 55 | 0.711 | no | Diphenhydramine+ | deep | Mixed intoxication | 36 |
| **ALC16** | m | 51 | 0.356 | yes, years | negative | n.m. | Intoxication | 143 |
| **ALC17** | m | 79 | 0.336 | no | negative | n.m. | Intoxication | 125 |
| **ALC18** | f | 49 | 0.575 | no | negative | n.m. | Intoxication | 52 |

**S1c Table.**  Characteristics of the deceased with alcohol-associated death.

PMI, postmortem interval; h, hours; ALC, alcohol; m, male; f, female; +, positive toxicological analysis; MOR, morphine; n.m., no measurement; deep; body core temperature <37.0 °C during postmortem examination.

|  | **Gender** | **Age** | **Survival time** | **Toxicological analyses** | **Cause of death** | **PMI (in h)** |
| --- | --- | --- | --- | --- | --- | --- |
| **TBI1** | m | 26 | 15 min | n.m. | Polytrauma | 108 |
| **TBI2** | m | 34 | 30 min | negative | Polytrauma | 120 |
| **TBI3** | m | 50 | 30 min | negative | Traumatic brain injury | 36 |
| **TBI4** | m | 73 | 5 h | n.m. | Traumatic brain injury | 68 |
| **TBI5** | m | 46 | 2 h | negative | Traumatic brain injury | 35 |
| **TBI6** | m | 24 | 30 min | negative | Polytrauma | 88 |
| **TBI7** | m | 74 | 8 h | negative | Traumatic brain injury | 108 |
| **TBI8** | m | 49 | 2 h 30 min | negative | Traumatic brain injury | 48 |
| **TBI9** | m | 25 | 4 h | negative | Traumatic brain injury | 38 |
| **TBI10** | m | 21 | 3.5 h | negative | Polytrauma | 117 |
| **TBI11** | m | 53 | 5 h | negative | Traumatic brain injury | 72 |
| **TBI12** | m | 25 | 2 h 30 min | *ALC 0.180 %* | Traumatic brain injury | 31 |
| **TBI13** | m | 41 | 40 min | *METH 733 ng/ml* | Traumatic brain injury | 42 |
| **TBI14** | m | 51 | 1 h 30 min | negative | Traumatic brain injury | 43 |

**S1d Table.**  Characteristics of the deceased with fatal traumatic brain injury (TBI).

PMI, postmortem interval; h, hours; m, male; min; minutes; n.m., no measurement; ALC; alcohol; METH, methamphetamine.

|  | **Gender** | **Age** | **Survival time** | **Toxicological analyses** | **Cause of death** | **PMI (in h)** |
| --- | --- | --- | --- | --- | --- | --- |
| **AMI1** | w | 75 | none | negative | Congestive heart failure | 94 |
| **AMI2** | m | 48 | 1 h 20 min | n.m. | Myocardial infarction | 68 |
| **AMI3** | m | 86 | 2 h | n.m. | Myocardial infarction | 119 |
| **AMI4** | m | 63 | none | negative | Coronary insufficiency | 154 |
| **AMI5** | w | 64 | none | negative | Congestive heart failure | 41 |
| **AMI6** | m | 75 | none | n.m. | Coronary insufficiency | 63 |
| **AMI7** | w | 73 | 1 h | negative | Congestive heart failure | 131 |
| **AMI8** | m | 49 | 50 min | negative | Myocardial infarction | 119 |
| **AMI9** | w | 49 | none | negative | Myocardial infarction | 28 |
| **AMI10** | m | 64 | none | negative | Congestive heart failure | 42 |
| **AMI11** | w | 71 | 3 h | *ALC 0.160 %* | Myocardial infarction | 108 |
| **AMI12** | m | 41 | 2 h 30 min | negative | Myocardial infarction | 59 |
| **AMI13** | m | 50 | none | negative | Coronary insufficiency | 117 |
| **AMI14** | m | 56 | 2 h | negative | Myocardial infarction | 111 |
| **AMI15** | m | 78 | 1 h 40 min | negative | Myocardial infarction | 97 |

**S1e Table.**  Characteristics of the deceased with acute myocardial injury (AMI).

PMI, postmortem interval; h, hours; min, minutes; n.m., no measurement; ALC, alcohol; m, male; ALC; alcohol.
